# Liposomal Formulations for Nose-to-Brain Delivery: Recent Advances and Future Perspectives

**DOI:** 10.3390/pharmaceutics11100540

**Published:** 2019-10-17

**Authors:** Soon-Seok Hong, Kyung Taek Oh, Han-Gon Choi, Soo-Jeong Lim

**Affiliations:** 1 Department of Integrated Bioscience and Biotechnology, Sejong University, 209 Neungdong-ro, Gwangjin-gu, Seoul 05006, Korea; kekeman1004@naver.com; 2 College of Pharmacy, Chung-ang University, 84 Heukseok-ro, Dongjak-gu, Seoul 06974, Korea; kyungoh@cau.ac.kr; 3 College of Pharmacy, Hangang University, 55 Hanyangdaehak-ro, Sangnok-gu, Ansan 15588, Korea; hangon@hanyang.ac.kr

**Keywords:** liposomes, intranasal, formulation, brain delivery, nanoparticle

## Abstract

Restricted drug entry to the brain that is closely associated with the existence of the blood brain barrier (BBB) has limited the accessibility of most potential active therapeutic compounds to the brain from the systemic circulation. Recently, evidences for the presence of direct nose-to-brain drug transport pathways have been accumulated by several studies and an intranasal drug administration route has gained attention as a promising way for providing direct access to the brain without the needs to cross to the BBB. Studies aiming for developing nanoparticles as an intranasal drug carrier have shown considerable promise in overcoming the challenges of intranasal drug delivery route. This review gives a comprehensive overview of works having investigated liposomes as a potential vehicle to deliver drugs to the brain through nose-to-brain route while considering the excellent biocompatibility and high potential of liposomes for clinical development. Herein, studies are reviewed with special emphasis on the impact of formulation factors, such as liposome composition and surface modification of liposomes with targeting moieties, in addition to intranasal environmental factors that may affect the extent/site of absorption of intranasally administered, liposome-encapsulated drugs.

## 1. Introduction

A number of patients suffering from neurodegenerative diseases and other central nervous system (CNS) disorders has shown a continuous increase that is associated with the aging population during the last decade. Alzheimer’s disease (AD), Parkinson’s disease, schizophrenia, migraine, malignant glioma, vestibular schwannoma, meningitis, and multiple sclerosis are representative CNS-related diseases [1]. However, effective therapeutic agents to treat most of these diseases are still missing, mainly due to the complicated, multifactorial pathogenic mechanisms [2]. Another major hurdle in the treatment of CNS diseases is the very limited accessibility of most potential active therapeutic compounds to the brain from the systemic circulation, which results in drug levels reaching the target site being insufficient to exert the pharmacological effect [3]. 

Restricted drug entry to the brain is closely associated with the existence of the blood brain barrier (BBB), the network of blood vessels that is comprised of tightly packed endothelial cells that separates the brain (central interstitial fluid) from the systemic circulation [4]. BBB has a selective permeability and it does not allow for the entry of harmful substances, such as toxins, bacteria, and chemicals. Only small lipophilic molecules can easily cross the BBB and almost all large molecular weight compounds (e.g., proteins) and over 98% of hydrophilic/low-molecular-weight drugs cannot cross the BBB, thereby suffering from the very low bioavailability in the brain [5]. 

Numerous attempts have been made to improve the drug delivery to the brain [6]. Strategies for facilitating drug transport across the BBB can be classified into invasive- and noninvasive ones. Invasive strategies include the chemical disruption of BBB by inducing temporarily shrinkage of endothelial cells, transient opening of tight junction, and allowing drug transport to the CNS [7,8]. Noninvasive strategies take advantage of the endogenous nutrient transport mechanisms that BBB has for the drugs penetrating the BBB [9,10,11], including transcytosis mediated by transporters expressed on the BBB. Numerous studies have shown that the conjugation of drug molecules or drug-entrapped vehicles to ligands against the transporter facilitates the active transport of drugs via specific interaction between ligands and transporters [12]. 

The intranasal administration route has gained interest as a promising alternative, providing direct access to the brain circumventing needs to cross the BBB [2,5]. Evidence for direct nose-to-brain transport pathways has been accumulated by a number of studies: a number of hydrophilic, high molecular-weight peptides/proteins appeared rapidly in the brain after intranasal administration and they reached in brain levels exceeding those that were obtained after intravenous administration [13]. For example, vasoactive intestinal peptide, interferon beta 1β, nerve growth factor, and insulin-like growth factor-I rapidly achieved peak brain levels exceeding those that were seen after intravenous administration in animals and humans [14]. Moreover, pharmacological activities of nasally administered peptides and proteins have been documented, including the memory improvement by intranasal insulin in healthy adults, obese men, and AD patients [15,16,17]. Several potential advantages of intranasal administration have also been proposed from these studies. They include the easy accessibility of the nasal cavity allowing for self-administration, improved patient compliance, rapid onset of action, minimized systemic exposure and reduced potential peripheral side effects, raising a possibility of clinical development of a dosage form with multiple benefits, in addition to an improved drug bioavailability in the brain [2,18]. 

Recent progress in nanotechnology has brought the development of nanoparticles as an advanced carrier delivering drugs [19,20,21,22]. These nanoparticles include liposomes [23,24], polymeric nanoparticles [25,26], solid lipid nanoparticles [27,28], micelles [29,30], nanostructured lipid carriers [31,32], nanoemulsions [18,33], and gold nanoparticles [34]. In a field of brain-targeted drug delivery, drug encapsulation in nanoparticles, after systemic administration, conferred drugs receptor- or adsorption- mediated transcytosis or endocytosis mechanism to cross the BBB, thereby increasing the amount of drugs reaching the target site in the brain [35,36]. Nanoparticle encapsulation has also increased the brain delivery of drugs after intranasal administration, which appears to be closely associated with increased stability, uptake, and extended residence time of drugs in the nasal cavity/epithelium [28,37,38,39]. Particularly, a significant portion of the research has been made focused on liposomes. This is due to the excellent biocompatibility, capability to load both hydrophilic and hydrophobic drugs, easiness of surface modification for targeting or increasing nasal residence time, and the high potential of liposomes for clinical development [9]. Investigation of liposomes as a potential vehicle to deliver drugs to the brain via BBB has been extensively reviewed elsewhere [1,10,24], and herein we will give an overview of liposome development as a carrier for nose-to brain delivery of drugs, based on the promising potential of nose-to-brain route for the brain targeting.

The present review summarizes the transport mechanisms by which nasally administered drugs gain access to the brain first, followed by a comprehensive overview of the latest progress in the field of liposome formulation as an intranasally administered carrier. Finally, implications and future perspectives will be given to better understand and give insight in developing liposomal formulations for nose-to-brain drug delivery. 

## 2. Drug Transport Route from the Nose to the Brain

**Figure 1:**
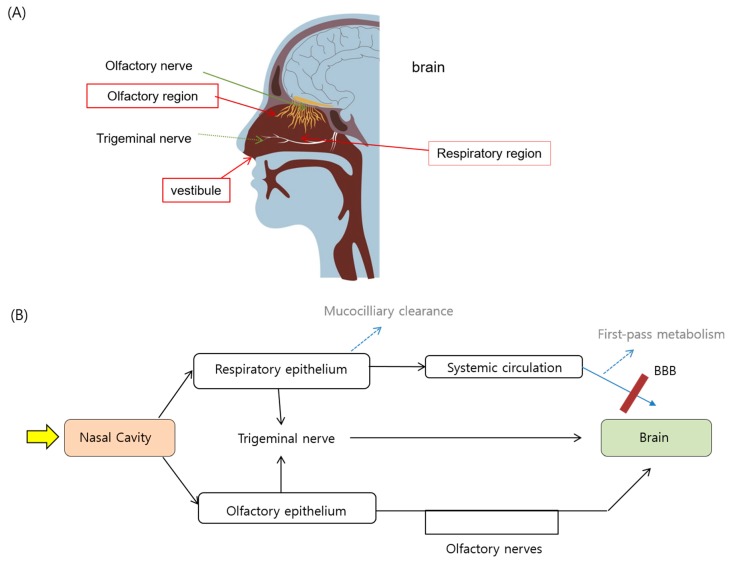
(**A**) Intranasal structure involved in the possible drug transport and (**B**) the potential drug transport routes leading to brain uptake following intranasal administration.

Despite growing interest, the mechanisms underlying the direct nose-to brain pathway of drug transport are not yet fully elucidated. Nevertheless, it is generally believed that there are direct and indirect pathways for drug transport from the intranasal cavity into the brain [40,41]: one is that drugs are directly transported into the brain via the olfactory or trigeminal pathways, and the other is that drugs reach the brain after first being absorbed into the systemic circulation and then crossing the BBB [42]. For better understanding of the nose-to-brain transport route, the anatomical structure of the nasal cavity (the interior of the nose) that might affect the nasal drug absorption would be briefly summarized first (Figure 1A), and the currently known mechanisms underlying the drug travelling routes to reach the brain after intranasal administration would then be reviewed (Figure 1B). 

### 2.1. Anatomy and Physiology of Nasal Cavity

The principal physiological functions of nose are the regulation of sense of smell (olfaction), allowing for air to enter the respiratory system and removing large particles, including pathogens from the inhaled air. Human nasal cavity has a total volume of 15~20 mL and a total surface area of 150~160 cm^2^ [38,43]. The epithelium of the nasal cavity is covered with a layer of mucosa. The mucus, together with cilia on the surface of ciliated cells, is the primary defense mechanism inside the nose by providing the mucociliary clearance: inhaled pathogens and particles are first entrapped in mucus and then dragged to the nasopharynx following the cilia movement, which results in clearing harmful substances within the nasal cavity every 15 to 20 min [44].

Nasal cavity can be divided into three regions, which are the nasal vestibule, the olfactory, and the respiratory regions (Figure 1A). The vestibular region is located immediately at the nostril openings [40]. The absorption of drugs in this region is the least important among three regions due to the small surface area (approximately 0.6 cm^2^) and the non-cilliated epithelium of the cell surface. Respiratory region occupies the largest part of the nasal cavity and the respiratory epithelium covering the respiratory region has the large surface area (~130 cm^2^) due to the presence of cells that possess numerous microvilli. This large surface area, combined with the high density of blood vessels, makes the respiratory region the major site of drug absorption into the systemic circulation if drugs are able to pass through the mucus layer to reach the surface of the epithelium. Respiratory region is also innervated by the trigeminal nerves, which extends from the brain stem and appear to be a potential target nerve for transporting drugs to the CNS [3]. 

The olfactory region is located in the deep upper part of the nasal cavity under the cribriform plate, the horizontal bone separating the nasal cavity from the brain. Cribriform plate has high perforations that provide access for the nerve endings to enter and, hence, constitute the unique part of the CNS that directly connects to the outside environment [5,43]. Olfactory epithelium consists of three types of cells, namely the basal cells, supporting cells, and the olfactory neural cells. Olfactory neurons extend from the olfactory bulb in the brain to the apical surface of the olfactory epithelium, with their cilia extending towards the nasal cavity (Figure 1A). The olfactory region in humans occupies 2~12.5 cm^2^ [2], corresponding to the 1.25~10% of the total surface area of the nasal cavity. Drugs passage from the nose to the brain through the olfactory epithelium might occur by diverse pathways, which will be reviewed in the next section.

### 2.2. Drug Transport Route 

If intranasally administered, drugs survive the mucociliary clearance in the vestibular region, move to the posterior regions of nasal cavity and contact with respiratory epithelium in the respiratory region. Some of them are absorbed through the epithelium into the blood or the lymphatic system, being subsequently transported into the systemic circulation. This pathway mainly accounts for the transcellular delivery of intranasally administered small lipophilic compounds. However, they need to cross the BBB to reach the brain, and they are still associated with hepatic drug metabolism and increased systemic exposure [2]. 

When drugs pass the vestibule and reach the respiratory epithelium, some of them may be transported thorough the branches of trigeminal nerves by being internalized to peripheral trigeminal neurons by endocytosis, being delivered afterwards to the brain stem and other connected structures, including the hindbrain and the forebrain. Although the contribution of the trigeminal nerve pathway in the direct nose-to-brain transport is evaluated to be smaller when compared to the olfactory pathway, the axonal transport of IGF-1 and interferon-β-1b through the trigeminal nerves has been reported, which confirms the existence of trigeminal nerve pathway [2,45]. 

**Figure 2:**
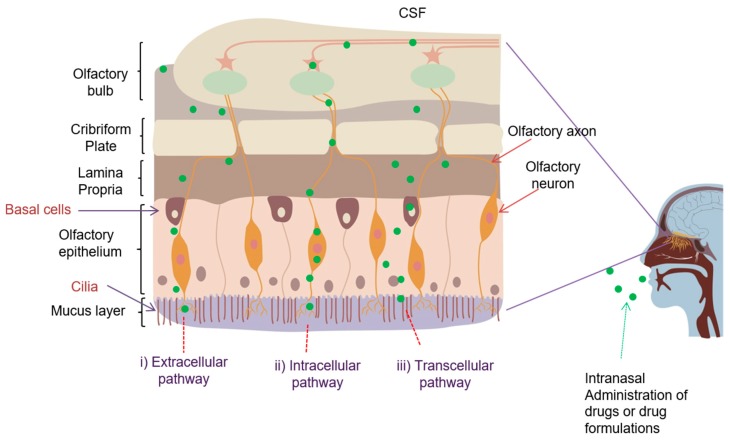
Schematic representation of various possible mechanisms involved in direct nose-to-brain drug transport from the olfactory region.

A small portion of the drug will be likely to reach the innermost olfactory region, where they can be transported to the brain by three different routes: i) intracellular (neuronal) transport pathway after internalization of drugs into the neurons; ii) extracellular transport across the spaces between cells, particularly along the channels near the olfactory nerves; and, iii) transcellular transport across the basal epithelial cells. Drugs or drug formulations following the intraneuronal pathway may enter the olfactory sensory neurons by endocytosis or pinocytosis, and are transported along the neurons into the olfactory bulb, where they are released by exocytosis and are further distributed to the different brain regions (Figure 2) [2]. Intraneuronal transport of drugs is relatively slow (hours to days), as shown in case of insulin growth factor-I and wheat germ agglutinin conjugated polyethylene glycol-polylactic acid nanoparticles [45,46]. In contrast, drugs transported through the extraneuronal pathway require only several minutes to reach the olfactory bulb and other brain regions. Much faster transport as compared to other pathways indicates the extracellular transport as the dominant pathway leading to the direct nose-to-brain transport. The leakiness that is caused by the co-existence of mature and newly formed neurons in some parts of the olfactory epithelium due to the slow regeneration of the olfactory neurons [47], in combination with the bulk flow of the cerebrospinal fluid into the brain along the length of neuronal axon, enables this direct, extracellular transport route to the drugs [47]. Lamina propria of the olfactory region, which is located beneath the epithelial layer, contains a neuronal supply that consists of olfactory axon bundles, presenting major extracellular pathways for drug transport. The transcellular route provides the drugs to lamina propria, which further enter the brain through passive diffusion or active transport, and it is mainly responsible for the transport of lipophilic molecules (Figure 2).

As depicted in Figure 1B and Figure 2, nasally administered drugs could reach the brain through different pathways; direct or indirect ones. Pathways leading to direct nose-to-brain transport could also occur through a single pathway or a combination of different pathways, according to the drug properties, formulation, and other physiological factors.

## 3. Role of Formulations in the Nose-to-Brain Drug Transport

### 3.1. Factors Affecting the Drug Transport

One of major factors determining the effectiveness of nose-to-brain drug transport is the physiological and environmental condition of intranasal cavity. The presence/activity of metabolic enzymes, including epoxide hydrolase, aldehyde hydrogenase, carboxylesterase, and glutathione S-transferase, in the nasal mucosa may affect drug metabolism [48,49]. Inconsistency of nasal mucosa properties such as tonicity and pH (generally 4.5~6.5) affected by physiological and environmental conditions may exaggerate the effect [50]. 

The effectiveness of nose-to-brain drug transport mainly depends on the physicochemical properties of drugs, such as the size, lipophilicity, and degree of ionization [5]. Drug properties, such as a high molecular weight, poor nasal mucosa penetration capacity, easy enzymatic degradation, and rapid mucocilliary clearance, result in a low total amount of drug transport into the brain [51]. Besides, the nasal absorption effectiveness is affected by the pH, osmolality, drug concentration, viscosity, surfactant, and physical state of the dosage form [5]. For instance, the pH of formulation can affect the drug stability and degree of the ionization, and it might provoke nasal mucosal irritation. Hyper- or hypotonic formulation may interfere with cilia movement, thereby affecting drug absorption. More viscous dosage forms may enhance contact them with the nasal mucosa, but might also decrease drug diffusion [35]. In addition to the administration device (e.g., spray or drop), administered volume and the head position during administration may also affect the extent and localization of drug deposition within the nasal cavity [35]. Supine position was shown to be advantageous in increasing the chance for the drug to reach the olfactory region. The optimal administrable volume of the intranasal administration is 5 μL for mice and 50 μL for rats (one side), while the human nasal cavity generally retains up to 200 μL [52]. Accordingly, the small administration volume currently limits the nasal formulation to potent drugs [53].

Among varying transport routes, the predominance of one pathway over the others principally depends on the physicochemical properties of drugs. The transcellular route is mainly responsible for the transport of lipophilic molecules. The extracellular route is suitable for the transport of hydrophilic drugs/substances, including proteins and peptides, allowing for the prompt onset of action, and it does not require the drugs’ binding to any receptors or undergoing axonal internalization. Small hydrophilic drugs (<1000 Da) may have rapid access to cerebral fluid compared to lipophilic drugs, since the former is less likely to be transported via the neuronal and supporting cells of the olfactory epithelium after nasal application. 

### 3.2. Role of Formulations in Nose to Brain Drug Transport

Several formulation approaches have been made to address the limitations of intranasal delivery. The limited drug absorption across the nasal epithelium and low transport to the brain tissues can be improved with permeation enhancers [54]. However, the potential toxicity of permeation enhancers after repeated use has limited their clinical use [55]. Mucoadhesive polymers, owing to their ability to interact with the mucus layer, may avoid the fast mucocilliary clearance and prolong the olfactory contact time [56]. A formulation that is able to protect drug from nasal enzymes, such as particle encapsulation, might reduce the enzymatic degradation of drugs in the nasal cavity, thereby improving its transport to the brain [37]. 

Nanotechnology-based formulations have the advantage of protecting the therapeutic cargo, in addition to improving and/or extending its interaction with the olfactory region [2,44]. Nanoparticles containing drugs may be endocytosed by the neurons and supporting cells in the olfactory region by a number of endocytic mechanismas and then drugs released, although nanoparticles that are larger than 100 nm are thought to have a restricted access to the intraneuronal route as their diameter exceeds that of the axons in the filia olfactoria [44]. Either the drug can be released from the nanoparticles in the nasal mucus layer from where it would be absorbed paracellularly or transcellularly through the epithelium. According to Huang and Donovan’s study, nanoparticles with relative hydrophilic surfaces tend to pass through the aqueous paracellular route, whereas those with hydrophobic ones are mainly transported via the transcellular pathway [57]. 

Nanoparticle-based formulations can be combined with enzymatic inhibitor, nasal absorption enhancers, and mucoadhesive polymers. Liposomes are spherical vesicle structures with an internal aqueous core that is surrounded by a single or multiple lamellar lipid bilayer [58]. Among different nano-based delivery systems, liposomes have gained considerable attraction because they are biocompatible [59], nontoxic [60], and capable of delivering both hydrophilic and lipophilic drugs [61]. Several liposome-based pharmaceutical products have been successfully translated into clinical use [62]. Nasal dosage form is not available yet and, at present, there are still a limited number of works for the development/application regarding liposome formulations for the direct nose-to-brain delivery. The next section will provide an extensive overview of literatures published in this field.

## 4. Strategies for Enhancing Nose-to-Brain Drug Transport by Using Liposomes

Liposomes for the intranasal administration have been developed for both hydrophilic and lipophilic drugs since hydrophilic drugs can be entrapped in the aqueous core of liposomes while lipophilic drugs inserted into the phospholipid bilayer surround the core [63]. After intranasal administration, lipophilic compounds may be indirectly transported to the brain by crossing the BBB after being systemically absorbed, as well as being directly transported to the brain via olfactory or trigeminal nerve pathways. In contrast, nasally administered hydrophilic and high molecular weight compounds would mainly use the direct pathway since they cannot cross the BBB, even if they are systemically absorbed through the nasal epithelium. With this difference in mind, recent works with hydrophilic drugs or lipophilic/amphiphilic drugs were summarized are Table 1 and Table 2, respectively.

### 4.1. Liposomes for the Nasal Delivery of Hydrophilic Drugs

The first four works listed in Table 1 investigated the effectiveness of nose-to-brain delivery of relatively large hydrophilic drugs (1000~45,000 M.W.). Migliore and coworkers performed the pioneering work with ovalbumin [66]. In this work, ovalbumin was entrapped in liposomes, whose surface was positively charged by stearylamine incorporation in the membrane. After nasal administration, a higher concentration of ovalbumin in brain was found in a liposome group as compared to a solution group, with negligible systemic absorption in both groups. The highest ovalbumin level in the brain was eight-fold higher in the liposome group, while the time to reach the C_max_ was similar (1-h post-administration) in both groups. Ovalbumin level in the brain remained higher up to 24-h, which thereby resulted in eight-fold higher AUC_0–infinity_ in rats receiving liposomes. Authors speculated that liposomes, particularly cationic liposomes, increased the residence time of ovalbumin at the olfactory epithelium, resulting in higher brain uptake. This research group also investigated the nasal delivery of glial-derived neurotrophic factor (GDNF)-encapsulated liposomes [64,65]. As in the case of ovalbumin, the highest GDNF level in the whole brain was obtained at 1-h post-administration. Among various brain sections, the GDNF level was particularly high in the section, including olfactory bulb, a site the closest to the administration site. However, at 24-h post-administration of either liposomes or solutions, very low GDNF level was detected in the brain. The neurotrophic and neuroprotective effects of intranasally administered GDNF were found, particularly after multiple dosing, but there was no difference between solution- and liposome groups. It is plausible that the lack of prolongation of GDNF residence time in the brain despite of liposome encapsulation, in contrast to the prolonged ovalbumin residence in the brain, resulted in similar therapeutic effects by GDNF.

Zheng and coworkers investigated the effectiveness of nasal delivery of β-amyloid protein breaker H102 peptide as a liposome formulation [68]. They first studied the differences in systemic pharmacokinetics: at 5 min after intravenous administration of H102 solution containing BSA and chitosan (as an absorption enhancer), H102 was undetectable in plasma, in accordance with the reported short plasma half-life (2 min) [68], while both H102 solution and liposome administered intranasally were detected at the same time point. Between H102 solution and liposomes intranasally administered, systemic T_max_ of liposomal H102 was slower than that of solutions (30 min vs. 5 min), which suggests the slower systemic absorption by liposome encapsulation. Systemic C_max_ of liposomal H102 was slightly higher than that of solution, while liposomal H102 stayed longer in the systemic blood circulation than H102 solution. After intravenous administration, H102 was undetectable in the brain, which indicated that H102 peptide cannot permeate the BBB. In contrast, nasal administration of solutions or liposomes led to significant brain uptake of H102, particularly at a high level in the olfactory bulb among four brain sections (cerebrum, cerebellum, hippocampus, and olfactory bulb). T_max_ of H102 in all brain sections of liposome group was slower than that of solution group and AUC_0-90min_ of H102 in a liposome group was higher than that of solution group (1.6~2.9-fold), which suggests a longer retention of liposomal H102. Authors explained the higher drug concentration over the time in various brain regions obtained by liposome encapsulation might be mainly attributed to the ability of liposomes to protect the H102 from enzymatic degradation. In this study, the therapeutic effect of H102 was also examined in an AD model in rat. Intranasally, but not intravenously, administered H102 significantly exhibited AD therapeutic activity in terms of spatial learning/memory and acetylcholine esterase activity, although no significant difference was found between the solutions and liposomes intranasally administered. This work highlights the effectiveness of liposomal encapsulation of H102 peptide in terms of increasing the brain uptake and residence time. 

More recently, Zhao and coworkers investigated the potential of nasal delivery of liposomal basic fibroblast growth factor (bFGF) in treating ischemic stroke [67]. The brain distribution of bFGF was compared at one time point (90 min after administration): bFGF level in all the brain sections was found to be minimal when bFGF was intravenously administered as a solution or a liposome formulation, which indicated that bFGF is not capable of crossing BBB. In contrast, bFGF was detected in the brain after intranasal dosing: liposomes gave higher bFGF level in all four brain sections (olfactory bulb, hippocampus, pallium, and striatum), with statistical significance in hippocampus and pallium, as compared to the solutions. After three consecutive day nasal treatments of liposomal bFGF to a stroke model rat, both neurological severity scores and spontaneous locomotor activities were improved more as compared to those of the solution. Together, this study gives direct evidence of the effectiveness of liposomes as an intranasal carrier to deliver BBB-impermeable drug to brain. Nevertheless, the formulation advantages shown in this study seem likely to be the combination of advantages of both polymeric nanoparticles and liposomes, as bFGF was indirectly encapsulated in liposomes after being first encapsulated in a gelatin nanoparticle core [67]. 

The next four works listed in Table 1 investigated the effectiveness of direct nose-to-brain delivery of hydrophilic drugs with a molecular weight less than 1000. Pashirova and coworkers first investigated the difference in the brain distribution of a fluorescent dye rhodamine as liposomes or solutions after intranasal or intravenous dosing (at 45 min post-administration) and found rhodamine at a high level in the cerebral cortex section only in the nasally administered liposome groups [69]. They next investigated the therapeutic effect of intranasally administered liposomal pyridine-2-aldoxime methochloride (2-PAM), an acetylcholine esterase reactivator, in a poisoned rat, in comparison with 2-PAM solution. Liposomes, but not solutions, were found to be therapeutically effective [69]. Ferric ammonium citrate, a drug for treating iron deficiency, was encapsulated in liposomes for intranasal dosing in Guo and workers’ study [71]. Liposomes or solutions were given to rats twice a day for one week and at one time point (24 h later of the last treatment) the brain level of ferric ammonium citrate was determined. As compared to solution, liposome increased the ferric ammomium citrate level higher in all brain sections (olfactory bulb, cerebellum, hippocampus, striatum, and cerebral cortex), particularly in the olfactory bulb section, which is a point of drug entry into the brain [71]. Li and coworkers encapsulated galanthamine hydrobromide (GH), a drug that is used to treat AD disease and ascular demenrtia, in liposomes [72]. Brain AUC_0→10h_ and C_max_ of drug determined after four-day administration (once a daily) of liposome formulation were significantly higher than those of free drug solution. Acetylcholine esterase activity in the brain determined after 10-day treatment was the highest in the case of intranasally administered liposomes, followed by intranasal solutions and oral solutions, demonstrating the highest AD therapeutic potential of nasal liposomal formulations [72]. Yang and coworkers investigated the potential of liposomal nasal delivery of rivastigmine, an AD drug that is currently available as an oral dosage form [70]. Liposomal formulations were compared with solutions containing poloxamer 188 as a permeation enhancer. Authors first studied the difference in the plasma rivastigmine concentration at 15, 60, and 240 min after intravenous or intranasal administration and they found (i) the intravenously administered solution group showed the lowest drug level in the plasma, which seems likely to be associated with rapid kidney uptake of drugs, and (ii) the intranasally administered solution group showed higher drug level and accordingly higher plasma AUC than intranasally administered liposome group. When the brain distribution of rivastigmine as liposomes or solutions was compared at 15, 60 and 240 min after intranasal administration, solutions exhibited higher drug level in olfactory bulb, hippocampus, and cerebrum section as compared to liposomes. T_max_ was similar (15 min) but C_max_ was higher and the duration was longer in the case of solutions. Enzymatic activities, pharmacodynamics markers of AD disease, were also more greatly increased in solution group than liposome group, which indicates the reduced therapeutic potential by liposome encapsulation [70]. 

Currently, the number of works reporting the application/development of liposomes for the intranasal delivery of hydrophilic drugs is very limited. In addition, experimental details, including a control group in animal models, are very different, which makes the strict comparison among studies difficult. For example, administration position, which is important for the intranasally administered drugs to reach the olfactory region, was described as supine [64,66,67,70,71], prone [72], or not described in some studies [68,69]. Despite these limitations, it is concluded that liposome encapsulation of hydrophilic drugs, particularly with high molecular weights, tend to increase the brain drug uptake in terms of concentration and duration time. Only one work that was done by Yang and coworkers [70] reported the inferiority of liposomes compared to solutions in terms of C_max_ and duration time of drug in the brain. With this regard, the use of permeation enhancer (poloxamer 188) in the rivastigmine solution, which was shown to enhance the rivastigmine absorption through nasal mucosa earlier [73], may be the cause of relative high brain uptake of intranasally administered rivastigmine solution. However, when considering that liposomal H102 formulation was more effective than the solution, despite the inclusion of permeation enhancer (chitosan) in the solution [68], the inferiority of liposomal rivastigmine cannot be fully attributed to the role of permeation enhancer. Interestingly, Migliore reported that reducing the liposomal dosing volume increased the direct nose-to-brain delivery of drugs [66]. The intranasal administration volume that was described by authors or obtained by our calculation based on the administration dose were 25~45 μL liposomes in most studies (two nostrils combined, [64,66,67,70,71,72]), except approximately 80 μL liposomes in case of rivastigmine [70]. Accordingly, it is plausible that too large dosing volume might cause most of the administered dose drained out of the nasal cavity into the esophagus, being the reason showing the underperformance of liposomal formulation. 

### 4.2. Liposomes for the Nasal Delivery of Lipophilic/Amphiphilic Drugs

Table 2 lists the works reporting the development of liposomal formulation of lipophilic or amphiphillic drugs. These drugs are also small (<500 M.W.) and they are expected to be easily diffused through nasal epithelium, and then being absorbed to the systemic circulation via transcellular pathway and permeating the BBB to reach the brain [1]. Therefore, their brain level obtained after intranasal administration would be the sum of direct/indirect drug transport to the brain.

Donepezil, an AD drug, was entrapped in liposomes, and the systemic and brain PK of liposomal donepezil were investigated after intranasal administration [74]. In the plasma, higher AUC, higher C_max,_ slower T_max_, and similar plasma half-life of donepezil were obtained in liposome group rats as compared to free drug (solution) group rats, whereas in the brain, higher AUC (twice), higher C_max,_ slower T_max_, and longer half-life was obtained in liposome group, which indicates that the liposomal formulation of donepezil was effective in increasing both the brain and systemic bioavailability of the drug. When the T_max_ of donepezil was compared between the plasma and brain, the brain T_max_ was slower than the plasma T_max_ (1.5 h vs. 1 h for liposomes and 1 h vs. 0.5 h for solution), suggesting that direct and indirect pathways both contribute to the donepezil transport to the brain regardless of liposomal encapsulation [74]. 

Hoekman and coworkers (2014) explored the potential of liposomal formulation of fentanyl citrate, an opioid analgesic. Authors prepared RGD (Arg-Gly-Asp) peptide-attached liposomes and used pressurized olfactory drug delivery device for intranasal administration of liposomal fentanyl [82]. In the systemic circulation, fentanyl in liposome formulations exhibited lower C_max_, slower T_max_, decreased AUC_0–120min_ compared to solutions. In the brain, fentanyl level was determined at only one point (5 min post-dosing) and no significant difference was found between free or liposomal fentanyl groups. In terms of analgesic effect, a slower onset was observed, but the effect lasted longer in liposome group when compared to free drug group. Overall, this work indicates a reduced systemic absorption of fentanyl and a slower brain uptake by liposomal encapsulation. Liposomes might function as a nasal depot slowly releasing drugs due to the preferential accumulation of RGD-modified liposomes at olfactory regions [75]. 

Narayan and coworkers [77] investigated the potential of liposomal encapsulation of a schizophrenia drug risperidone in rats. Authors prepared three different formulations of liposomes with slightly anionic (soyPC:CHOL), cationic (SPC/CHOL/SA), or highly anionic SPC:CHOL:DSPE-PEG) surface charges, while the particle sizes were similar (Table 2). Regardless of liposomal compositions, intranasal administration of liposomes resulted in lower C_max_ and slower T_max_ (30 min vs. 5 min) in the systemic circulation as compared to intravenously administered free drug. However, plasma AUC (AUC_0–t_ & AUC_0–inf_) of risperidone increased in a liposome group, indicating the sustained systemic absorption of the liposomal drugs through the nasal cavity. Among three liposome formulations, DSPE-PEG-containing liposomes exhibited the highest plasma AUC, probably due to the long circulation of liposomes by PEGylation. In the brain, intranasal administration of liposomes resulted in higher C_max_, higher AUC and faster T_max_ as compared to solution that was administered by the same route (15 min vs. 60 min). DSPE-PEG-containing liposomes showed the highest AUC and the longest brain residence time among liposomal formulations. The ratio of AUC_brain_ to AUC_plasma_ of DSPE-PEG-containing liposomes, but not those of other liposomal formulations, was larger than 1, indicating the maximized brain bioavailability by encapsulating in PEGylated liposomes. This work implies the followings: the brain transport of significant portion of these drugs might occur through systemic absorption of the drug across the BBB when the lipophilic/amphiphilic drugs are encapsulated in liposomes. Therefore, PEGylation of liposomes, which can extend the systemic circulation of liposomes, might be advantageous to increase the brain transport of BBB-permeable drugs, like risperidone after intranasal administration. This work also evaluated the drug targeting efficiency (% DTE), a well-known parameter assessing the overall tendency of the drug to accumulate in the brain following intranasal administration vs. intranvenous administration ([AUC_brain_/AUC_plasma_ *IN*]/[AUC_brain_/AUC_plasma_ *IV*]) of three liposome formulations [35]. The DTE of DSPE-PEG-containing liposomes was found to be higher than 100% and the authors speculated that the long-circulation of liposomes highly contributed to increasing the overall uptake of risperidone into the brain, which indicated the direct nose to brain transport route of risperidone. Guo and coworkers [76] studied the potential of liposomal formulation of celecoxib as an AD drug in mice. Celecoxib is a very lipophilic drug known to pass the BBB, but prone to rapid metabolism [83]. The plasma C_max_ of intranasally administered liposomal celecoxib was higher than that of free drug (aqueous suspension) administered by the same route. The increased celecoxib concentration by liposome encapsulation seems likely to be at least partly due to the improvement of the solubility limitation of celecoxib by liposomal encapsulation. When the brain level of intranasally administered celecoxib was determined by fluorescence analysis of celecoxib-quantum dot (CB-QD) conjugate or by LC-mass analysis of celecoxib, liposomal drug was detected at much higher levels, from the earlier time points and for the longer period as compared to free drug. The liposome group and free drug group both exhibited the faster T_max_ in the brain when compared to that in the systemic circulation. Importantly, the neuronal uptake of celecoxib was found, regardless of formulations and separate experiments that were done with cultured neuronal cells demonstrated that the liposome encapsulation facilitates the intraneuronal uptake of CB-QD. Collectively, the increased brain transport of celecoxib by liposomal encapsulation appears to involve increased nose-to-brain transport via intraneuronal pathway. In accordance with the brain pharmacokinetic data, the aggregation of amyloid β protein in the brain of disease model mice was more effectively reduced by three-month administration of liposomes than that of free drugs, which demonstrates the more potent therapeutic effect of liposome formulations [76]. 

Liposomal formulation of quetiapine fumarate, a schizophrenia drug, for the intranasal administration, was studied in a mice model [79]. The brain distribution of liposomes was first determined by gamma scintigraphy of ^99m^Tc and liposome-entrapped ^99m^Tc exhibited higher brain accumulation when compared to free ^99m^Tc dispersion. When the quetiapine level in the brain homogenate was determined by HPLC analysis, it was found that liposomal drug was detected at much higher levels, from the later time points and for the longer period as compared to free drug, which indicates increased brain bioavailability of quetiapine by liposome encapsulation. Based on data, the authors proposed the gain of an alternative brain entry pathway for the intranasally administered liposomal quetiapine (e.g., endocytosis). 

In other study, olanzapine was encapsulated in liposomes [78]. SDC or Span60 was included in the liposome membrane as an edge activator for increasing the elasticity of liposomes. Intranasally administered liposomes exhibited lower AUC_0–360min_, lower C_max_, and faster clearance of olanzapine in the systemic circulation as compared to intranasally administered free drug solution. Plasma T_max_ and C_max_ of drug obtained in intranasally administered SDC-containing liposome group was slower and much lower, while those of the Span60-containing liposome group were similar to those of the free drug group. In the brain, all three formulations exhibited similar T_max_ and mean residence times, but the intravenously administered free drug group exhibited the highest AUC_0–360min_ and C_max_. Between two liposome formulations, Span60 liposomes showed higher C_max_ and AUC_0–360min_ than SDC liposomes. In combination with in vitro liposome deformability data, the authors speculated that the liposomes with better deformability, by being able to be squeezed through the nasal mucosa easier, showing higher bioavailability in both the plasma and the brain. The authors also speculated that the minimal effectiveness of liposomal formulation of olanzapine might be due to the intrinsic high permeability of free drug crossing the nasal layer and BBB easily [84].

Most works listed in Table 2 demonstrated the increased C_max,_ prolonged drug distribution in the brain and longer duration of therapeutic effects by liposomal encapsulation [72,74,75,76,77,79]. Liposomes appear to help in overcoming the solubility limitations of lipophilic drugs and function as an intranasal drug depot slowly releasing these drugs. Liposomes also seem likely to provide an alternative direct brain transport pathway (e.g., endocytosis of liposomes) to these drugs. In addition, the systemic circulation half-life of BBB-permeable drugs might be extended by liposomal encapsulation, thereby further increasing the drug amount reaching the brain indirectly after passing BBB. However, differences in experimental details among the listed studies should still be mentioned. For example, studies were done in mice [76,79] or in rats [74,75,77,78]. Anatomical differences among species may affect the nasal blood flow, enzymatic activity, mucociliary clearance, and intranasal pH [51], thereby affecting nasal drug adsorption. Administration positions were different: supine [79], prone [78], or not described [74,75,76,77]. Lowered brain C_max_ by liposomal encapsulation of olanzapine shown by Salama [78] in opposite to increases in most studies [74,76,77,79] may be at least partly associated with the opposite administration position taken in Salama’s work. The application of olfactory delivery-specialized device using liposome aerosol was attempted in one study [75], and it might result in much preferential drug accumulation in the olfactory region as compared to other works, in which drugs were applied without a specialized device. Dosing volumes were also very different (20 μL (rat, [75]), 50 μL (rat, [77]), and 100 μL (mice, [79]) or not described [74,76,78]. As a control group, free drugs were intravenously administered [77,78], or intranasally administered [74,75,76,79], to make strict comparison among studies more difficult. In the case of very lipophilic drugs, a simple dispersion of a free drug, instead of solution, was used in the control group and, in these cases, the increased brain transport of drugs that were obtained by liposomal encapsulation would reflect the capability of liposomes to improving the poor aqueous solubility of drugs together [76,79]. Ex vivo transport studies using sheep nasal mucosa were done for an AD drug tacrine and an epilepsy drug lamotrigine [80,81] and their works demonstrated an increased nasal permeability of drugs by liposomal encapsulation. Data that were obtained by performing in vivo studies with these drugs would be helpful in drawing a solid conclusion regarding the potential of liposomal formulation for nose-to-brain delivery of lipophilic/amphiphilic drugs. 

### 4.3. Pharmaceutical Considerations in Developing Liposomal Nasal Formulations 

It is well known that the physicochemical characteristics of nanoparticles (e.g., size and surface charges) are a critical factor affecting the various properties of nanoparticles as a drug carrier [2]. In a research field of developing nanoparticles as a nasal formulation, literatures have demonstrated the impact of surface charge on the nasal residence time of drugs/nanoparticles, and it appears that cationic particles tend to exhibit increased nasal residence time due to electrostatic interactions between nanoparticles and the mucous layer [51]. With this in mind, several works, as listed in Table 1 and Table 2, made the surface charge of liposomes positive by inserting cationic lipid [56,64,66,77] or cationic surfactant [69] between phospholipid molecules, or additional coating of liposomes with mucoadhesive polymers [85,86]. Surface positivity in these studies might further contribute to increasing the brain drug uptake through increasing/extending the drug accumulation over the olfactory region. In addition, loading liposomes in a mucoadhesive gel also appears to provide a more extended nasal residence time [56]. 

From literatures, the sizes of nanoparticles should be smaller than 50 nm to penetrate the nasal epithelium via extracellular routes [57,75]. Another study investigating the brain distribution of nanoemulsions after intranasal administration demonstrated that ~100 nm nanoemulsions were transported along the olfactory and trigeminal pathway, whereas larger emulsions were not [87]. In contrast, other studies have reported the effective size of nanocarriers up to 200 nm, which appears to be associated with the average diameter of olfactory exons (around 200 nm) [1]. The impact of liposome sizes on the direct nose-to-brain delivery has not yet been reported. Nevertheless, it is noteworthy that most works that are listed in Table 1 and Table 2 used liposomes with sizes in the range of 70~300 nm and these liposomes still exerted effects increasing drug delivery to the brain, although some differences exist among studies, as discussed in previous sections. In one study, liposomes that are even larger than 400 nm still showed the tendency to increase the brain uptake of encapsulated drugs [78]. It may be associated with the better flexibility of liposomes (e.g., membrane fusion leading to endocytosis) compared to other nanoparticles. With this regard, several recent works used transferosome, a modified liposome in which edge activators (e.g., Tween 80, Span 70 or SDC) were inserted between phospholipid molecules [69,72,78,81]. Further increased flexibility/deformability of liposomes provided by edge activators might enable the liposomes to squeeze through the nasal mucosa more easily, which thereby raises the upper size limit that was observed in other nanoparticles’ cases. 

Surface modification of liposomes is known to increase the liposome binding to target sites or the systemic circulation half-life of encapsulated drugs [31,49]. Surface attachment of cell-penetrating peptide [70] or RGD [75] or PEGylation of liposomes [68,77] was attempted in some studies. However, the effectiveness of surface modification was not fully evaluated in these studies and, hence, further studies of formulation scientists are required to address how much it contributes to the brain bioavailability of drugs, with simultaneous consideration of the drug properties. For example, drugs that are incapable of crossing BBB following the systemic circulation, PEGylation may not be an effective way to increase the total drug uptake into the brain. 

In a study that was performed with positively- or negatively charged polymeric nanoparticles, anionic nanoparticles showed a preference for the olfactory pathway, whereas cationic ones did for trigeminal pathways [1], which suggests the possible role of surface charge of nanoparticles in determining the major drug transport pathway reaching brain. Collectively, compositions and/or surface modification of liposomes appear to be critical factors determining the efficacy of liposome formulations for the nose-to-brain drug delivery. Further studies, including ex vivo studies in a nasal mucus layer model and in vivo studies in an animal model, are warranted for addressing the impact of liposomal formulation factors on the brain delivery more clearly.

Earlier literatures have demonstrated that the addition of permeation enhancers in the drug solution is effective in increasing the nasal absorption of poorly permeable drugs by transiently inducing tight junction opening between epithelial cells [54]. However, the use of permeation enhancers has been limited due to their nasal toxicity [55]. The signs of nasal toxicity after intranasal administration of liposomes were found to be minimal in several studies [68,70,79,81]. This raises the possibility of developing optimized liposomes as a safe, nontoxic nasal drug carrier for intranasal administration. 

## 5. Conclusion: Implications for Future Development

Studies for developing nanoparticle formulations aiming at enhancing the nose-to-brain drug delivery have shown considerable promise for the future. Among various nanoparticles, liposomes have gained attraction as a formulation to deliver drugs to the brain by the nasal route due to the high potential of liposomes for clinical development. However, a limited number of works has still been reported in the field of liposomal formulations for the nose-to-brain delivery. Nevertheless, works that were reported over the last years indicate the effectiveness of liposomes as a nose-to-brain carrier of drugs, particularly hydrophilic and macromolecular ones. Works suggest that the optimization of formulations parameters, such as phospholipid composition, insertion of edge activators, PEGylation, surface modification of liposomes with targeting moieties, and/or combination of liposomes with other carrier systems, such as in-situ gelling hydrogel, would contribute to further enhancing the drug bioavailability in the brain. In doing optimization studies, the impacts of environment liposomes would meet after nasal administration should be simultaneously considered. For example, the changes of the tonicity of the nasal environment may considerably affect the liposomal formulation stability and the drug-releasing rate [51]. Formulations that can minimize the particle aggregation at nasal pH would be required to ensure the intranasal stability of liposomes, as cationic liposomes are prone to aggregation in physiological conditions [88]. Setting reliable in vitro or ex vivo study models that can test the impact of these formulation factors would be essential for facilitating the development of liposome formulations for nose-to-brain delivery. More detailed and elaborate experimental settings would be helpful in evaluating the impact of liposomal formulation on % DTE and % DTP (% nose-to-brain direct transport) of drugs. Formulation strategies for improving the drug loading capacity of liposomes would also be warranted, as the drug should be encapsulated in liposomes at a concentration high enough to be nasally administrable in a few microliters. Mechanistic studies for distinguishing which pathways predominate in terms of the extent of drug transport to the brain, which is also affected by the formulation factors of liposomes, would be required for providing solid evidence on the contribution of the factors. Comprehensive studies aiming to develop an optimal liposomal formulation for the nose-to-brain drug delivery may shed light on opening new opportunities for treating CNS-related diseases in the future. 

## Figures and Tables

**Table 1:** Liposomal formulations designed for nose-to-brain delivery of hydrophilic drugs.

Composition	Particle Size	Zeta Potential	Drug (MW)	Target Disease	In Vivo	Dosing Position	Dosing Volume	Ref.
DOPC:CHOL:SA	149 nm	+30 mV	GDNF (15,100)	Parkinson’s Disease	SD rat	supine	25 μL	[64,65]
DOPC:CHOL:SA	299 nm	+19 mV	ovalbumin (43,000)		SD rat	supine	25 μL	[66]
HSPC:CHOL	128 nm	−15 mV	bFGF (16,500)	ischemic stroke	SD rat	supine	~30 μL	[67]
EPC:CHOL:DSPE-PEG	112 nm	+3 mV	H102 peptide (1,289)	AD	SD rat		40 μL	[68]
soyPC:DHAHAB	142 nm	+6 mV	2-PAM (173)	Organoph-osphorous poisoning	Wistar rat			[69]
EPC:CHOL	166 nm	+11 mV	rivastigmine tartrate (400)	AD	SD rat	supine	~80 μL	[70]
EPC:CHOL	40 nm	−48 mV	ferric ammonium citrate (262)	iron deficiency	SD rat	supine	45 μL	[71]
soyPC:CHOL (30:0.2)	112 nm	+49 mV	galanthamine hydrobromide (368)	AD	SD rat	prone	40 μL	[72]

1,2-dioleoyl-sn-glycero-3-phosphocholine (DOPC); cholesterol (CHOL); *N*-(Carbonyl-methoxypolyethyleneglycol 2000)-1, 2-distearoyl-sn-glycero-3-phosphoethanolamine (DSPE-PEG); egg-phosphatidylcholine (EPC); stearylamine (SA); glial-derived neutotrophic factor (GDNF); dihexadecylmethylhydroxyethylammonium bromide (DHAHAB); hydrogenated soy phosphatidylcholine (HSPC); basic fibroblast growh factor (bFGF); pyridine-2-aldoxime methochloride (2-PAM).

**Table 2:** Liposomal formulations designed for nose-to-brain delivery of lipophilic/amphiphilic drugs.

Composition	Size	Zeta Potential	Drug (MW)	Disease	In Vivo	Dosing Position	Dosing Volume	Ref.
DSPC:CHOL:PEG	102 nm	−28 mV	donepezil (379)	AD	Wistar rat			[74]
DMPC:DMPG	104 nm	NA	fentanyl citrate (336)	opioid analgesic	Rat	POD	20 μL for PK	[75]
Phospholipid:CHOL	126 nm	−9 mV	celecoxib (381)	AD	Mice			[76]
soyPC:CHOL, soyPC:CHOL:SA or soyPC:CHOL: DSPE-PEG	90~100 nm	−54, +15, −29 mV	risperidone (410)	Schizophrenia	Wistar rat		50 μL	[77]
l-α-PC:SDC or l-α-PC:Span60	380~410 nm	NA	olanzapine (312)	Schizophrenia	Wistar rats	Prostrate		[78]
EPC:CHOL	152 nm	+25 mV	quetiapine fumarate (384)	Schizophrenia	albino mice	Supine	100 μL	[79]
EPC:CHOL:α-tocopherol:Omega	192 nm	−15 mV	tacrine hydrochloride (235)	AD				[80]
phospholipid90G:CHOL:Tween80	140 nm	NA	lamotrigine (256)	Epilepsy				[81]

eggphosphatidylcholine (EPC); 1,2-distearyl-sn-glycero-3-phosphocholine (DSPC); cholesterol (CHOL); polyethylene glycol (PEG); 1,2-dimiristoyl-sn-glycero-3-phosphoglycerol (DMPG); *N*-(Carbonyl-methoxypolyethyleneglycol 2000)-1, 2-distearoyl-sn-glycero-3-phosphoethanolamine (DSPE-PEG), Omega; soyPC; pressurized olfactory drug delivery device (POD); sodium deoxycholate (SDC).
